# Acute Mental Stress is Associated With Altered Pupillary Light Reflex

**DOI:** 10.33549/physiolres.935518

**Published:** 2025-06-01

**Authors:** Zuzana VISNOVCOVA, Nikola FERENCOVA, Ingrid TONHAJZEROVA

**Affiliations:** 1 Biomedical Centre Martin, Jessenius Faculty of Medicine in Martin, Comenius University in Bratislava, Martin, Slovak Republic; 2 Department of Physiology, Jessenius Faculty of Medicine in Martin, Comenius University in Bratislava, Martin, Slovak Republic

**Keywords:** Pupillary light reflex, Acute mental stress, Cognitive processing, Parasympathetic nervous system, Psychophysiology

## Abstract

Pupillary light reflex (PLR), i.e. pupil constriction in response to light stimulus, offers a sensitive index of parasympathetic nervous regulation. Yet, the studies on PLR in acute mental stress are rare. We aimed to study potential changes of PLR to acute mental stress in healthy young people with respect to sex. Thirty-eight participants (24 women, age: 22.95±0.19 yrs) were examined in a cross-sectional study under standard conditions. PLR parameters were measured separately for both eyes using PLR-2000 (NeurOptics, USA) before arithmetic test (baseline), immediately after arithmetic test, and after recovery period. Evaluated PLR parasympathetically-mediated parameters: pupil diameter (mm) before (initial value, INIT) and after illumination (peak of constriction, END), maximum constriction velocity (MCV), relative constriction amplitude (RCA). INIT and END diameters were significantly reduced immediately after mental stress and after recovery compared to baseline (left eye: INIT: p=0.044, p=0.035, respectively; END: p=0.004, p<0.001, respectively; right eye: INIT: p<0.001, p=0.002, respectively; END: p<0.001 for both comparisons). No significant differences were found in MCV and RCA. Moreover, the study established no significant changes in the assessed PLR parameters between sex throughout the stress protocol. Our study revealed that acute mental stress is associated with greater PLR-parasympathetic response resulting in prolonged pupil constriction. This finding could represent the first step for understanding the effect of cognitive processing on PLR under physiological conditions, before its clinical application.

## Introduction

Pupil size is regulated by two antagonistic muscles – sphincter and dilatator in the iris. The contraction of the sphincter innervated predominantly by the parasympathetic nervous system leads to pupil contraction and *vice versa* – the dilatator innervated primarily by sympathetic nervous system evokes pupil dilatation. Importantly, the sympathetic branch is mediated by hypothalamic nuclei and the parasympathetic pathway is mediated by central inhibition of the Edinger-Westphal complex of the oculomotor nucleus in the midbrain [1]. Thus, the changes in pupil size as a result of stimulation of the dilatator or inhibition of the constrictor can provide important information related to central autonomic activity [2,3].

In this context, the autonomic nervous system plays a crucial role in the complex regulation of affective, cognitive and emotional functions indicating flexibility and adaptability in response to stress. The psychophysiological research focused on parasympathetic (vagal) functioning in stress response suggests that stress response could be predominantly determined by cardiac-linked vagal activity indexed by heart rate variability (HRV) [4]. Our previous studies revealed that short-term HRV analyzed by linear and nonlinear method is sensitive to various mental tasks [5–9]. From this perspective, pupillary light reflex (PLR), i.e. pupil constriction in response to illumination, depends mainly on parasympathetic nervous system. Specifically, the afferent pathway from the retinal receptors includes a synapse in the pretectum before secondary projections reach the motor center in the oculomotor nucleus representing the third parasympathetic nerve. The efferent pathway involves a synapse at the ciliary ganglion associated with final neurons synapsing on pupillary sphincter as the major component of pupillary constriction [10]. Thus, PLR evaluation could represent a promising noninvasive diagnostic tool for a wide range of clinical conditions providing important information about dynamic parasympathetically-mediated responses under central autonomic control [11].

Several studies indicated that pupil light response is involved in cognitive and emotional processing associated with attention. For example, PLR inhibition was demonstrated in anticipation of fear-provoking stimuli [12] or dynamic PLR parameters were changed to arithmetic task [10,13]. Recently, PLR was altered during a left-to-right comparison task with auditory stimuli suggesting the modulation of PLR *via* mechanisms of spatial attention operating on mental contents [14]. However, the effect of acute mental stress on dynamic parasympathetically-mediated PLR is still unclear. Moreover, the effect of sex on PLR also remains questionable and barely studied. While Filipe *et al*. [15] found no significant differences in PLR with respect to sex in young participants (age range: 14–33 years old), Fan *et al*. [16] revealed higher initial diameter of pupil under dark conditions in young men compared to young women (age range: 18–22 years old), but without significant sex-related differences under light conditions. In addition, recent study revealed that mental stress may accelerate parasympathetic activation indexed by PLR indices in males [13]. Thus, further research in this area is needed. Therefore, we aimed to study the potential PLR changes in response to mental arithmetic task for both eyes with respect to sex in healthy young subjects.

## Methods

The study was approved by the Ethics Committee of Jessenius Faculty of Medicine in Martin, Comenius University in Bratislava in accordance with the 1964 Helsinki declaration and its later amendments. All participants were carefully instructed about the study protocol and they gave written informed consent prior the examination.

### Subjects

We examined 38 healthy young undergraduate students (average age: 22.95±0.19 yrs, body mass index: 22.50±0.41 kg/m^2^; 24 women – average age: 22.90±0.13 yrs, body mass index: 21.10±0.41 kg/m^2^, and 12 men – average age: 23.00±0.48 yrs, body mass index: 24.80±0.40 kg/m^2^) in the psychophysiological laboratory at the Department of Physiology and Biomedical Centre Martin (BioMed) of Jessenius Faculty of Medicine in Martin. Following exclusion criteria were applied: underweight, overweight, obesity, history of cardiovascular, respiratory, endocrine, neurological, metabolic or infectious diseases, mental disorders, alcohol and psychoactive drugs abuse, smoking.

### Protocol

The examination was performed in a quiet darkened room with the same light intensity under standard conditions (temperature 22–23 °C, minimization of stimuli) between 01:00–03:00 p.m. The subjects were instructed to sit comfortably in a special armchair for 15 min necessary for adaptation to the examination conditions and elimination of the potential effects of stress. Then, pupillary light reflex indices were evaluated for both eyes using a handheld infrared optical scanner Pupillometer PLR-2000 (NeuroOptics, USA) with a sampling frequency 32 Hz and accuracy 0.1 mm. The subjects were asked to keep their head straight and keep both eyes wide open without blinking. The pupillometer was kept at a right angle to the subject’s axis of vision without tilting the device. The margins of the pupil were electronically detected under infrared illumination and tracked during 5 s of continuous recording of the pupil after application of light stimulus with intensity of 180 mW and duration 154 ms. The examiner was seated across from the subject in the identically tall chair as the subject was seated in the special armchair across with a table of an appropriate height between them to ensure the stability of the pupillometer holding during the measurement. Each eye was measured separately always in the same order (left eye was measured first). The subjects rested from 30 s to 60 s before measuring the second eye [17]. It is important to note that monocular measurement is not associated with biasing the results of the second eye [18]. The PLR was examined in the following order: before arithmetic test (basal period) – immediately after arithmetic test – at the end of the examination (recovery phase). Each phase lasted 2 min (according to [13]).

### Mental arithmetic test

**Fig. 1:**
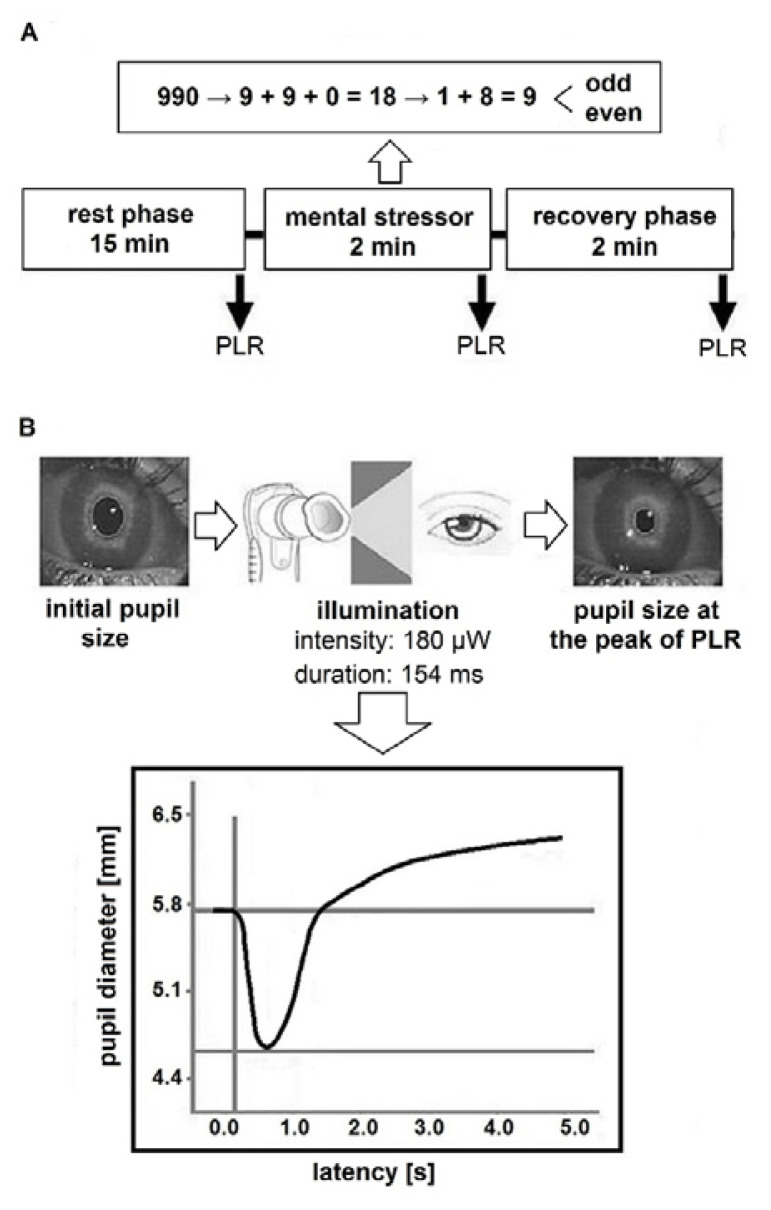
(**a**) The study protocol. (**b**) The continuous recording of pupil diameter in response to light stimulus.

Mental arithmetic test is considered as a standard medium-intensity psychophysiological stressor [19] that causes a shift in autonomic dynamic balance towards parasympathetic underactivity (vagal withdrawal) associated with sympathetic hyperactivity. The subjects summated individual digits of three-digit numbers displayed on the monitor into one-digit number. Consequently, volunteers decided if the result was even or odd by pushing the arrows on keyboard (even-right, odd-left). The study protocol is shown in Figure 1.

### Evaluated PLR Parameters

We focused on the evaluation of parasympathetically-mediated PLR parameters [11,20]. Specifically, the pupil diameter in millimeters was assessed before the application of light stimulus (initial value, INIT) and after illumination at the peak of the constriction (final value, END) in each phase of stress protocol. Furthermore, maximum constriction velocity (MCV, mm/s) and relative constriction amplitude (RCA, %) representing the constriction percentage change of the pupil diameter [11] were evaluated. The recording of pupillary light reflex is shown in Figure 1.

### Statistical analysis

The data were analyzed using statistical software package SYSTAT (Cranes Software International Ltd., USA). The normality of distribution was assessed using Shapiro-Wilk test and the equality of variance using Levene’s test. MCV, INIT and END pupil diameter showed normal distribution and equal variances across the evaluated samples. The effects of the individual stress-related phases of the protocol (baseline vs. stress vs. recovery), the sex and the side differences (left vs. right eye) were assessed using two-way repeated-measures analysis of variance (ANOVA) and *post hoc* Fisher’s Least-Significant-Difference test. Since RCA was not normally distributed, the effects of the individual stress-related phases of the protocol (baseline vs. stress vs. recovery), the sex and the side differences (left vs. right eye) were analyzed using Kruskal-Wallis and Wilcoxon test. The probabilities p<0.05 were considered as significant. The data are expressed as mean ± SE.

## Results

ANOVA revealed a significant effect of the individual stress-related phases of the protocol for both INIT and END pupil diameter (F_[2]_=7.006, p=0.002; F_[2]_=13.553, p<0.001, respectively). No significant differences were found in the PLR parameters relative constriction amplitude (RCA, χ^2^_[2]_=1.830, p=0.401) and maximum constriction velocity (MCV, F_[2]_=1.668, p=0.196).

### The left eye

**Fig. 2:**
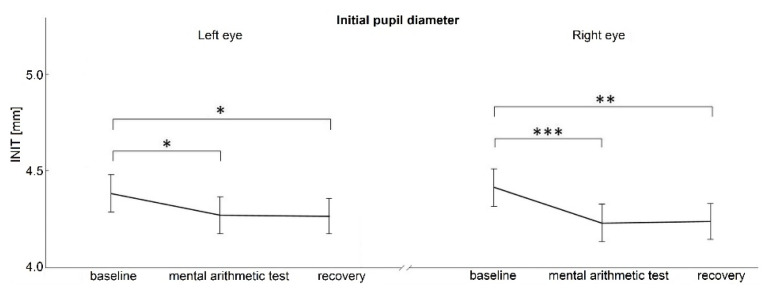
Initial pupil diameter before the application of light stimulus. * p<0.05, ** p<0.01, *** p<0.001. INIT, initial pupil diameter; mm, millimeters.

**Fig. 3:**
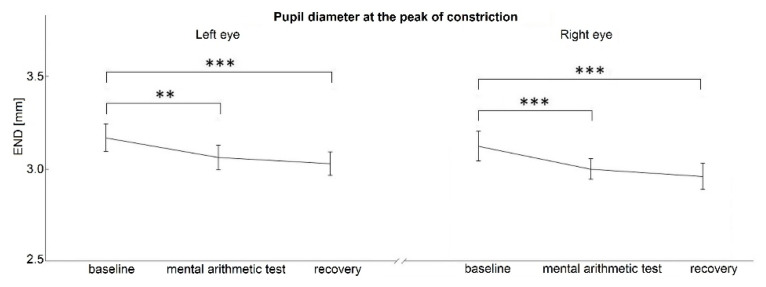
Pupil diameter at the peak of constriction after the application of light stimulus. ** p<0.01, *** p<0.001. END, pupil diameter at the peak of constriction; mm, millimeters.

*Post hoc* analysis revealed that both PLR parameters – initial pupil diameter before light application (INIT) and after light illumination at the peak of the constriction (END) were significantly reduced in the immediate phase after mental arithmetic stress and at the end of examination (recovery phase) compared to basal phase (INIT: p=0.044, p=0.035, respectively; END: p=0.004, p<0.001, respectively; Figs 2, 3).

### The right eye

*Post hoc* analysis revealed that both PLR parameters – initial pupil diameter before light application (INIT) and after light illumination at the peak of the constriction (END) were significantly reduced in the immediate phase after mental arithmetic stress and at the end of examination (recovery phase) compared to basal phase (INIT: p<0.001, p=0.002, respectively; END: p<0.001 for both comparisons, Figs 2, 3).

### Comparison between the left and right eye

Statistical analysis revealed no significant differences in the evaluated parameters between the left and right eye within the whole duration of protocol.

### Comparison between the sex (women vs. men)

No significant differences were found in the evaluated parameters between the sex during all phases of examination protocol.

## Discussion

In this study, we assessed resting and activated central autonomic activity using pupillary light reflex in response to mental stress in young healthy students. The findings of this study revealed that mental stress significantly affected the PLR pattern and its reactivity to light stimulus resulting in ongoing pupil constriction after arithmetic test mediated primarily by parasympathetic nervous system. It seems that discrete alterations in central autonomic integrity evoked by mental stress could be associated with attention and cognitive processing. Several explanations are assumed. The pathway for the pupillary light reaction provides a promising tool to investigate psychological influences associated with varying modes of activation. Specifically, the primary circuit for the pupillary light reflex is localized at subcortical level, i.e. the Edinger-Westphal nucleus receiving excitatory input from the pretectal olivary nucleus enabled by retinal ganglion cells [21,22]. Moreover, the pupil response to light is innervated by neural networks implicated in physiological arousal and executive functioning including subcortical as well as cortical brain structures. Recent studies noted that the alerting network is innervated by the noradrenergic system and includes the locus coeruleus, frontal and parietal cortex. From this perspective, noradrenaline plays a key role in cortical system stimulation resulting in adequate central activation for cognitive performance. Thus, pupillary responses can reflect the activity of the complex central network important for psychological processes [2,23].

The enlarged pupillary diameter – *dilatation* – could be mediated by greater sympathetic activation and/or greater inhibition at the Edinger-Westphal center. Notably, the activity of parasympathetic oculomotor center is inhibited by the sympathetic-linked noradrenergic system involving locus coeruleus [24] and it is modulated by inhibitory effects from cortical pathways [1,2]. We suggest that our finding related to resting greater pupillary diameter compared to reduced pupillary diameter in the phases after stress might reflect increased sympathetic activity as a result of anticipatory anxiety or increased physiological arousal before psychological stress. In particular, arousal phenomenon is mediated by complex central structures including reticular activating system. The increased reticular activation primarily inhibits the Edinger-Westphal complex, thus, any increment in psychosensory stimulation or other central activation such as anticipation could have immediately resulted in increased pupil diameter [10]. Importantly, another study concluded that the resting pupil size is more strongly affected by the sympathetic nervous system [13]. We suggest that complex neural processing including adaptation to the dark before application of cognitive test can result in the larger pupil diameter and more extensive pupil response.

Furthermore, the dynamic pupil response to light was characterized by ongoing decreased pupillary diameter – *constriction* – immediately after mental stress and in recovery phase indicating potential effect of cognitive process on parasympathetically-mediated PLR responses. In this context, Steinhauer *et al*. [10] found reduced extent of the phasic light reaction in mental arithmetic test in healthy volunteers suggesting that the locus of interference with the light was the Edinger-Westphal complex of the oculomotor nucleus associated with descending cortical inhibitory influences of the Edinger-Westphal complex. In contrast, our results of the prolonged pupillary constriction after mental stress in healthy students could indicate sympathetic withdrawal associated with reduced inhibition on the oculomotor complex resulting in brisk shift of dynamic autonomic balance towards parasympathetic activation. It is important to note that vagal “rebound”, i.e. rapid increase in parasympathetic activity after stress is an indicator of flexibility and adaptability of the organism [9,25]. Further, decreased physiological “arousal” characterized by parasympathetic overexcitation associated with sympathetic underactivity could represent an important mechanism contributing to pupil constriction in the phases after stress.

Besides physiological variations in PLR, several studies have pointed out that pathological conditions such as neurological disorders including Alzheimer’s and Parkinson’s diseases [26–29] and mental health disorders including major depressive disorder, generalized anxiety disorder, attention-deficit/hyperactivity disorder, autism spectrum disorder [3,30–33] can alter the PLR compared to healthy subjects. These findings can indicate the application of PLR examination as a helpful tool in the management of a wide range of pathological conditions in clinical practice, especially in psychiatry, where objective diagnostic markers still lack.

## Study limitations

Potential limitation of this study is a relatively small homogeneous group of young healthy subjects. From this point of view, the findings of this study cannot be extrapolated to general population including older age and need to be independently validated in larger groups of people with respect to sex differences. Moreover, the pupillometric analysis provides different information of autonomic nervous system activity compared to other methods, such as electrodermal response or HRV [22]. Thus, complex evaluation of other distinct biosignals characterizing autonomic nervous system associated with PLR could reveal important information about central-peripheral pathway in mental stress. Further research in this field is needed.

## Conclusions

In conclusion, our study revealed that acute cognitive stress influenced the PLR pattern in the manner of greater parasympathetically-mediated response resulting in prolonged pupil constriction after stress. Thus, it could represent the first step for understanding of cognitive processing on the PLR in physiological conditions before the clinical application.
